# Changes of Ocular Dimensions as a Marker of Disease Progression in a Murine Model of Pigmentary Glaucoma

**DOI:** 10.3389/fphar.2020.573238

**Published:** 2020-09-04

**Authors:** Michał Fiedorowicz, Marlena Wełniak-Kamińska, Maciej Świątkiewicz, Jarosław Orzeł, Tomasz Chorągiewicz, Mario Damiano Toro, Robert Rejdak, Piotr Bogorodzki, Paweł Grieb

**Affiliations:** ^1^ Department of Experimental Pharmacology, Mossakowski Medical Research Centre, Polish Academy of Sciences, Warsaw, Poland; ^2^ Small Animal Magnetic Resonance Imaging Laboratory, Mossakowski Medical Research Centre, Polish Academy of Sciences, Warsaw, Poland; ^3^ Faculty of Electronics and Information Technology, Warsaw University of Technology, Warsaw, Poland; ^4^ Department of General Ophthalmology, Medical University of Lublin, Lublin, Poland; ^5^ Faculty of Medical Sciences, Collegium Medicum, Cardinal Stefan Wyszyński University, Warsaw, Poland

**Keywords:** glaucoma, ocular dimensions, ocular biomechanics, intraocular pressure, DBA/2J, MRI

## Abstract

**Purpose:**

The elevation of intraocular pressure (IOP), a major risk factor in glaucoma, is an important parameter tracked in experimental models of this disease. However, IOP measurement in laboratory rodents is challenging and may not correlate with some key pathological events that occur in the development of glaucoma. The aims of this study were to quantify changes in ocular morphology in DBA/2J mice that develop spontaneous, age-dependent, pigmentary glaucoma and to check the possible correlation of these parameters with IOP.

**Method:**

Eye morphology was evaluated with MRI in DBA/2J, DBA/2J-Gpnmb^+^/SjJ, and C57BL/6J female mice ages 3, 6, 9, 12, and 15 months. The animals were anesthetized with isoflurane. A planar receive-only surface coil (inner diameter = 10 mm) was placed over each animal’s left eye and the image was acquired with a 7T small animal-dedicated magnetic resonance tomograph and T2-weighted TurboRARE sequence. Ocular dimensions were manually quantitated using OsiriX software. IOP was measured with rebound tonometry.

**Results:**

In the control animals, no age-related changes in the ocular morphology were noted. Since 6 months of age, the anterior chamber deepening and elongation of the eyeballs of DBA/2J mice was detectable. We found a significant, positive correlation between IOP and axial length, anterior chamber area, or anterior chamber width in C57BL/6J mice but not in DBA/2J mice. However, after excluding the measurements performed in the oldest DBA/2J mice (i.e. analyzing only the animals ages 3 to 12 months), we demonstrated a significant positive correlation between IOP and anterior chamber width.

**Conclusion:**

High-resolution magnetic resonance imaging of the eye area in mice enables reproducible and consistent measures of key dimensions of the eyeball. We observed age-dependent alterations in the eye morphology of DBA/2J mice that mostly affected the anterior chamber. We also demonstrated a correlation between some of the ocular dimensions and the IOP of C57Bl/6J mice and DBA/2J mice with moderately advanced glaucomatous pathology.

## Introduction

Glaucoma is a heterogeneous group of chronic disorders characterized by a relatively selective, progressive loss of retinal ganglion cells and their axons which form the optic nerve. It is the most common cause of irreversible blindness worldwide, currently affecting almost 80 million people (Tham et al., 2014), or more than 1% of the global population. Ocular hypertension (OH) is a major and modifiable risk factor for the onset and progression of glaucoma. Lowering intraocular pressure (IOP), currently the only accepted method of glaucoma treatment, may be achieved pharmacologically, and in some cases surgically (Crawley et al., 2012; Lusthaus and Goldberg, 2019).

Treatments aimed at decreasing IOP are ineffective in many cases. Surgical intervention, considered the most effective procedure for lowering IOP in uncontrolled glaucoma with OH, is not available to the vast majority of patients in need; similarly, surgery is unavailable to more than three-quarters of patients suffering from vision impairment due to cataract or uncorrected refractive error, conditions that are fully treatable surgically (Flaxman et al., 2017). Moreover, glaucoma surgery is risky, because of various potentially dangerous complications (Giovingo, 2014). Pharmacological treatment, on the other hand, requires lifelong daily instillation of IOP-lowering eye drops, and this type of drug formulation suffers from particularly low long-time adherence and persistence (Yeaw et al., 2009). Considering the above, there is an urgent need to develop more efficacious methods for chronic IOP control.

The assessment of safety and efficacy of a new treatment with the use of animal models is usually a prerequisite for initiating the clinical development phase. The preclinical assessment of drugs aimed at lowering IOP has been traditionally performed on monkeys with artificially increased IOP (Wang et al., 2000), but such experiments are prohibitively expensive and currently rarely conducted due to ethical reasons. Instead, following the development of non-invasive technologies of IOP measurement applicable to small animals, IOP-dependent rodent models have become popular, in which OH is artificially induced (Millar and Pang, 2015; Biswas and Wan, 2019). Although these models reproduce certain aspects of human glaucoma, since retinal ganglion cell (RGC) loss does not develop naturally, an increase in IOP is usually abrupt and may not be long-lasting, and inflammatory components due to physical injury may be a confounding factor.

Few rodent models of IOP-dependent glaucoma have been described in which no surgical intervention is required and the disease develops spontaneously with age (Chen and Zhang, 2015). Among them, the most popular involves the DBA/2J strain of mice. Its predecessor, the original DBA strain initiated in 1909 at Harvard University, is the oldest of all inbred murine strains (Harada et al., 2019; Jackson Laboratory, 2020). DBA/2J mice develop progressive eye abnormalities that mimic human pigmentary glaucoma. The onset of disease symptoms begins between three and four months of age. Mutations in glycoprotein nonmetastatic melanoma protein B (*Gpnmb*) and tyrosinase-related protein 1 (*Tyrp1*) genes causing iris atrophy and iris pigment dispersion leading to elevated IOP, although OH is not the only causative factor (Anderson et al., 2006; Scholz et al., 2008).

In spite of the popularity of this model among investigators, the development of glaucoma in the DBA/2J mouse is highly variable, presenting signiﬁcant challenges for preclinical studies of anti-glaucoma therapies. In particular, IOP measurements may be confounded by factors such as corneal calciﬁcation, etc. (Turner et al., 2017). On the other hand, imaging techniques such as whole-eye optical coherence tomography (Chou et al., 2011) and dynamic, contrast-enhanced magnetic resonance imaging (MRI) (Crosbie et al., 2019) revealed characteristic, age-dependent changes of eyeball morphology in DBA/2J mice, including an increased anterior chamber depth. Such structural changes did not occur in the control C57Bl/2J mice; therefore they seem to stem from chronic OH. However, contributions from other genetic factors differentiating these two strains of mice cannot be excluded. Despite some available data on changes in ocular morphology in DBA/2J mice (Chou et al., 2011; Crosbie et al., 2019), the age-related changes in ocular morphology in the course of spontaneous glaucoma were not extensively characterized to date. In particular, the correlation between the ocular dimensions and IOP needs further investigation with imaging methods that allow *in vivo* evaluation of the whole eye globe. Such data would be crucial for future application of selected ocular dimensions as imaging markers of the glaucoma progression and effectiveness of IOP-lowering therapies in this model of glaucoma.

The primary aim of this study was to evaluate age-dependent changes in the ocular morphology in the course of glaucoma in DBA/2J mice. We also aimed to verify the hypothesis that parameters describing ocular morphology correlate with IOP. Age-matched DBA/2J-Gpnmb^+^/SjJ and C57BL/6J mice were used as controls.

## Materials and Methods

### Animals

All experimental procedures were performed in compliance with the ARVO Statement for the Use of Animals in Ophthalmic and Vision Research and in accordance with the provisions for animal care and use described in the European Communities council directive of September 22, 2010 (2010/63/EU) and respective local regulations for animal experiments; the experiments were approved by the respective local ethics committee (Warsaw IV Local Ethics Committee, approval nos. 88/2012, 94/2012, 11/2015). The experiment was performed on three groups: (1) DBA/2J model of glaucoma (n = 10); (2) C57BL/6J (n = 10; RRID : IMSR_JAX:000664) which were obtained from the murine breeding colony at the Mossakowski Medical Research Centre; and (3) the DBA/2J-Gpnmb^+^/SjJ mice (n = 8, RRID : IMSR_JAX:000664) which were obtained from Jackson Laboratory. Due to the fact that DBA/2J females develop iris atrophy and glaucoma earlier than males (John et al., 1998; Anderson et al., 2001), all mice used in this study were female. The same mice were examined at 3, 6, 9, 12, and 15 months of age. Mice were maintained in the Mossakowski Medical Research Centre animal house at 12 h-12 h light-dark cycle and were provided a standardized diet and water *ad libitum.*

### Administration of Manganese Chloride

To visualize the neural retina by the manganese-enhanced magnetic resonance imaging (MEMRI), a solution of manganese chloride (0.1 mol/L, Sigma Aldrich, Steinheim, Germany) was administered intraperitoneally (66 mg/kg body weight) 24 h prior to the imaging session (Calkins et al., 2008).

### Magnetic Resonance Imaging

Animals were anesthetized with isoflurane (4% of isoflurane in oxygen for induction, 2% in oxygen for maintenance); this concentration was chosen to minimize eye movements during the acquisition (Woodward et al., 2007). Subsequently, the animals were positioned on a dedicated mice scan bed (Bruker, Ettlingen, Germany), and a planar receive-only surface coil (inner diameter = 10 mm; Bruker BioSpin, Ettlingen, Germany) was placed over the left eye of each animal. A rodent-dedicated 7T magnetic resonance (MR) tomograph (BioSpec 70/30USR Bruker, Ettlingen, Germany) was equipped with a transmit coil (inner diameter = 86 mm, Bruker). Physiological monitoring, including respiration rate and body temperature, was performed throughout the imaging sessions with an MR-compatible system (SA Instruments, Stony Brook, NY, USA). Details of the imaging protocol included:

1. Structural, high-resolution images were acquired with a T2-weighted TurboRARE sequence (TR/TEeff = 2700/30 ms, RARE factor = 8, NA = 12, spatial resolution = 0.62 mm x 0.62 mm x 0.3 mm, slices = 7, no gaps, scan time 16 min);
2. Visualization of accumulated manganese (MEMRI) with FLASH T1-weighted 3D sequence (TR/TE = 48/4 ms, NA = 5, FA = 25°, spatial resolution 68 μm x 68 μm x 68 μm, scan time 28 min).

### Evaluation of Eye and Optic Nerve Dimensions

**Figure 1:**
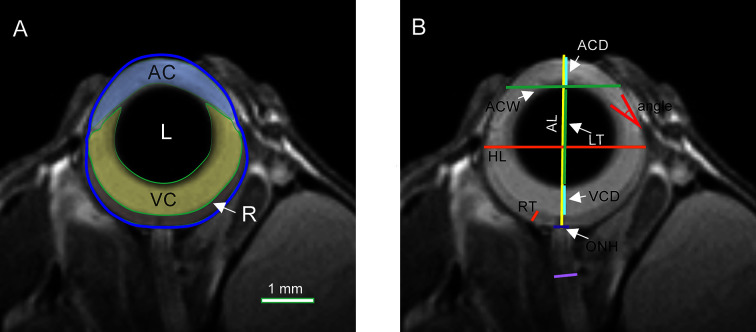
Quantification of ocular morphology. **(A)** Areas of anterior chamber (AC), lens (L), vitreous chamber (VC), and retina (R). **(B)** Dimensions: axial length (AL), equatorial diameter (ED), anterior chamber depth (ACD), anterior chamber width (ACW), lens thickness (LT), vitreous chamber depth (VCD), retinal thickness (RT), optic nerve head diameter (ONH), and optic nerve diameter (ON).

Ocular and optic nerve dimensions and areas were manually delineated and calculated with OsiriX software (version 5.8.2, 32 bit; Pixmeo, SARL, Bermex, Switzerland, RRID : SCR_013618) by a researcher unaware of the animal group or age. All measurements were performed on the middle image, i.e. the one with the visible optic nerve head and the largest pupil diameter (Chou et al., 2011). Areas of the anterior chamber (AC), lens (L), vitreous cavity (VC), and retina (R) were measured as shown in **Figure 1A**. The axial length (AL), horizontal length (HL), anterior chamber depth (ACD), anterior chamber width (ACW), lens thickness (LT), vitreous chamber depth (VCD), retinal thickness (RT [measured 0.5 mm from the optic disc center]), optic nerve head diameter (ONH), and iridocorneal angle were also measured (**Figure 1B**). The AL, ACD, LT, and VCD distances were appointed along the eye axial line passing through the center of the pupil and the center of optic nerve head. AL was established as the distance between the anterior cornea surface and the center sclera level of the intraocular optic nerve. The ED measurement was made at the widest place of the eye globe below the *ora serrata*. The RT was appointed as the distance between the nerve fiber layer and the retinal pigment epithelium (RPE).

The ACD was used to compute the corneal radius of curvature (CRC) according to the method described by Tkatchenko et al. (2010).

$$CRC=(ACD/2)+ACW^{2}/(8\;x\;ACD)$$

The first measurement of the optic nerve was performed on the ONH at the level of the sclera. The second was on the intraorbital segment of the optic nerve, below 0.8 mm behind the globe where myelinization of the nerve fibers starts (May and LüTjen-Drecoll, 2002).

### IOP Measurements

A rodent-dedicated rebound tonometer (TonoLab; Icare, Finland) was used for IOP measurements as we described previously (Fiedorowicz et al., 2018; Świątkiewicz et al., 2020). In brief, the probe tip was situated in the long axis of the eye at 1 mm to 2 mm distance from the eye. Six valid measurements were acquired and the result was calculated as the mean of four readings (after excluding two outlier readings), which was displayed as a single reading. The measurement procedure was repeated three times for each eye. The measurements were performed on conscious animals to avoid the influence of anesthesia on IOP (Camras et al., 2010). To reduce the animals’ stress during immobilization, habituation sessions took place on five consecutive days before the measurements. To exclude the influence of diurnal variations, all IOP measurements were performed at the same time each day (10 a.m. to 12 a.m.).

### Statistical Analysis

Statistical analysis was performed with Statistica for Windows software (version 10, StatSoft, Inc. Tulsa, OK, USA, www.statsoft.com, RRID : SCR_014213) and GraphPad Prism (version 8.3.0, GraphPad Software, San Diego, CA, USA, www.GraphPad.com, RRID : SCR_002798). In cases of morphometric parameters or IOP, a two-way ANOVA followed by a Dunnett multiple comparisons test or a Sidak’s test were used. MEMRI results were analyzed with Kruskal-Wallis non-parametric ANOVA followed by Dunn’s multiple comparisons *post hoc* test. Data are presented as mean ± standard deviation. Differences were regarded as significant for P < 0.05. Spearman’s rank correlation analysis was performed with GraphPad Prism. Correlations were regarded as significant for P < 0.05.

## Results

**Figure 2:**
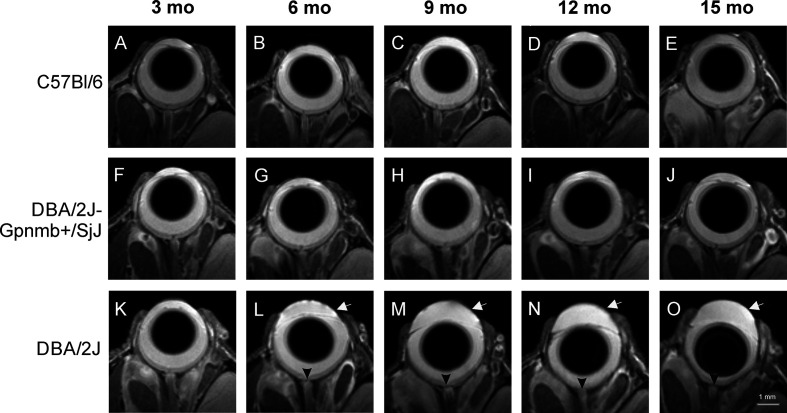
Age-dependent changes in eye morphology in C57BL/6J (first row, **A–E**), DBA/2J-Gpnmb^+^/SjJ (second row, **F–J**), and DBA/2J (third row, **K–O**) mice. DBA/2J mice display anterior chamber deepening (arrow) and optic disc cupping (arrowhead).

Anatomical images of the eye revealed no remarkable changes in the morphology of C57BL/6J (**Figures 2A–E**) or DBA/2J-Gpnmb^+^/SjJ mice (**Figures 2F–J**). No differences were observed between the 3-month-old DBA/2J mice and the animals from the other groups. However, from 6 months of age, deepening of the anterior chamber was clearly visible in DBA/2J mice (**Figure 2K–O**) as well as optic disc cupping.

**Figure 3:**
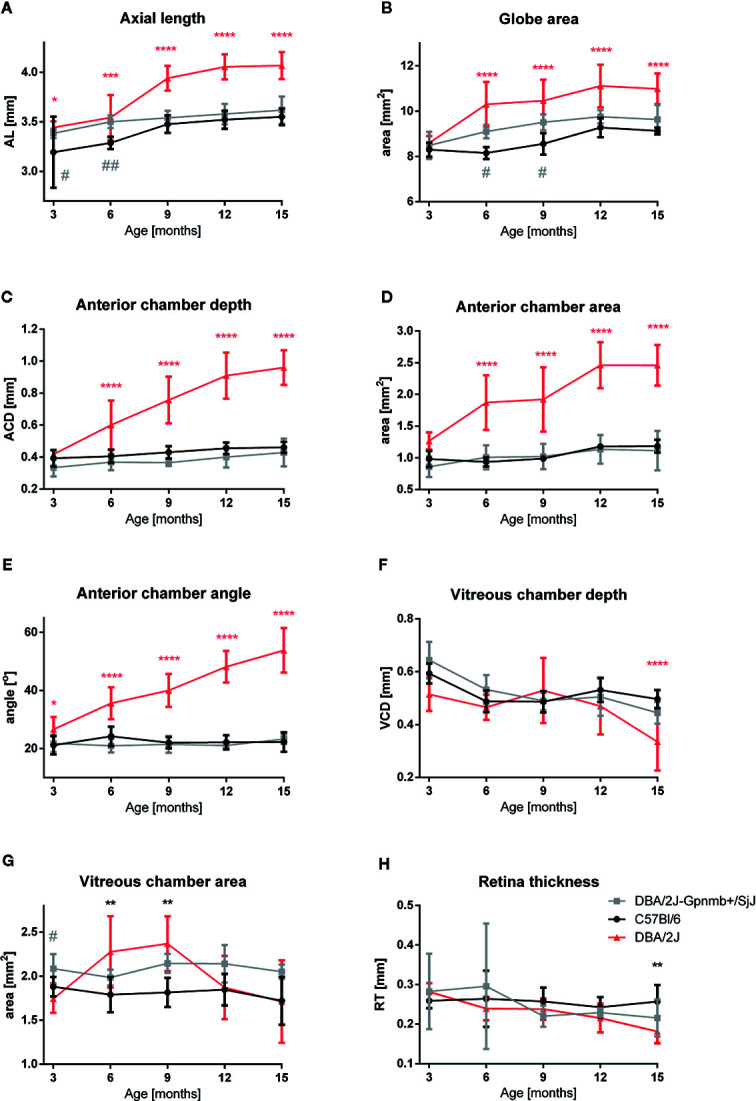
Quantitative analysis of eye morphology in C57BL/6J, DBA/2J-Gpnmb+/SjJ, and DBA/2J mice. **(A)** axial length, **(B)** globe area, **(C)** anterior chamber depth, **(D)** anterior chamber area, **(E)** iridocorneal angle, **(F)** vitreous chamber depth, **(G)** vitreous chamber area, and **(H)** retinal thickness. Means ± SD. *P < 0.05, **P < 0.01, ***P < 0.001, ****P < 0.0001 DBA/2J vs. C57BL/6J; ^#^P < 0.05, ^##^P < 0.01 DBA/2J-Gpnmb^+^/SjJ vs. C57BL/6J. Two-way ANOVA followed by Dunnet’s multiple comparisons test.

Quantitative analyses of these images confirmed these findings (**Figure 3**). No significant differences were noted in the ocular morphology between the control animals at any time. Parameters of the eye globe size in 3-month-old animals of different strains did not differ significantly: The eye globe area in DBA/2J was 8.592 ± 0.115 mm^2^; in DBA/2J-Gpnmb^+^/SjJ it was 8.490 ± 0.271 mm^2^; and in C57BL/6J it was 8.302 ± 0.113 mm^2^. However, from 6 months of age we noted anterior chamber deepening as well as enlargement and elongation of the eyeball in DBA/2J. Between the third and ninth months of age, the eye globe area increased by about 22% in DBA/2J, 12% in DBA/2J-Gpnmb^+^/SjJ, and 3.0% in C57BL/6J. The eyeball lengthened in DBA/2J mice with age. AL was slightly longer in 3-month-old DBA (3.442 ± 0.024 vs. DBA/2J-Gpnmb^+^/SjJ; P < 0.05; 3.383 ± 0.017 mm and C57BL/6J 3.193 ± 0.127 mm; P < 0.05) and from 6 months, AL significantly increased with age in DBA/2J mice. The most prominent elongation of AL in DBA/2J was from 6 months (3.542 ± 0.072 mm) to 9 months (3.939 ± 0.039 mm), compared with control mice (DBA/2J-Gpnmb^+^/SjJ at 6 months AL = 3.499 ± 0.022 mm; P < 0.0001 and at 9 months 3.539 ± 0.027 mm; P < 0.0001; C57BL/6J in 6 months had 3.285 ± 0.20 mm; P < 0.0001 and in 9 months 3.476 ± 0.028 mm; P < 0.0001).

Anterior chamber enlargement seemed to contribute mainly to AL elongation. No significant differences were noted in 3-month-old mice between strains in ACD (DBA/2J 0.418 ± 0.010 mm; DBA/2J-Gpnmb^+^/SjJ 0.334 ± 0.020; C57BL/6J 0.393 ± 0.018 mm) and in ACA (1.265 ± 0.052; 0.859 ± 0.072;.982 ± 0.045mm^2^; respectively). Thereafter, the ACD and ACA constantly increased in DBA/2J. The most pronounced ACD and ACA enlargement was in older DBA/2J mice. In 15-month-old DBA/2J mice, ACD was 0.961 ± 0.034 mm and ACA was 2.460 ± 0.134 mm^2^ vs. DBA/2J-Gpnmb^+^/SjJ ACD 0.429 ± 0.031 mm; P < 0.0001; ACA 1.113 ± 0.179 mm^2^; P < 0.0001 and C57BL/6J ACD 0.461 ± 0.011 mm; P < 0.0001; ACA 1.185 ± 0.046 mm^2^; P < 0.0001.

Similarly, we noted only a slight difference in the anterior chamber angle in 3-month-old animals (DBA/2J 26.620 ± 1.610⁰ vs. DBA/2J-Gpnmb+/SjJ 21.757 ± 1.323⁰; P < 0.05; P < 0.05; DBA/2J vs. C57BL/6J 21.163 ± 1.117⁰; P < 0.05). However, in older DBA/2J mice the angle constantly widened and in 15-month-old animals it reached 53.794 ± 2.714⁰. In the other strains, the angle remained stable (in DBA/2J-Gpnmb^+^/SjJ: 23.258 ± 0.631⁰; P < 0.0001 and in C57BL/6J: 22.256 ± 1.178⁰; P < 0.0001). However, no significant changes in CRC between strains were noted at any time.

The area of the vitreous chamber was higher in 9-month-old mice (2.369 mm^2^ ± 0.311, P < 0. 05) than in the 3-month-old DBA/2J mice (1.743 ± 0.159 mm^2^). In older animals, VCA was similar as in the 3-month-old mice. Such an increase was not observed in either of the control strains. However, the depth of the vitreous body significantly dropped between the twelfth and fifteenth months in DBA/2J mice (0.470 ± 0.108 mm vs. 0.334 ± 0.109 mm, P < 0.01). In the control strains, VCD remained relatively stable. We did not notice significant differences in the lens area between the strains at any time.

**Figure 4:**
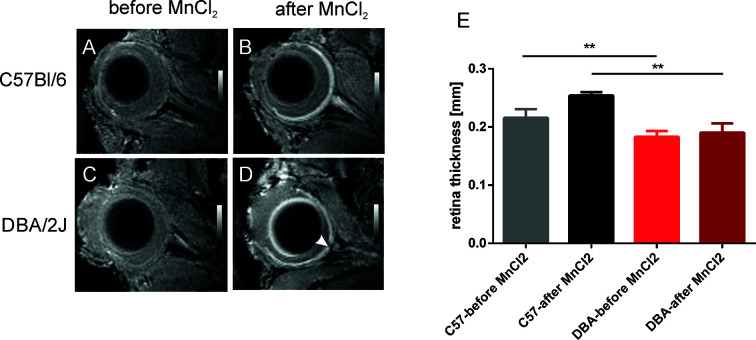
Visualization and quantification of the retina after systemic administration of manganese. Left panel shows representative T1-weighted images of the eyes before **(A**, **C)** and 24 h after **(B**, **D)** intraperitoneal injection of manganese chloride in C57BL/6J **(A**, **B)** and DBA/2J mice **(C**, **D)**. The arrowhead indicates optic disc cupping in DBA/2J mice. **(E)** Results of quantification of retina thickness in these mice before and after MnCl_2_ administration. **P < 0.01; Kruskal-Wallis non-parametric ANOVA followed by Dunn’s multiple comparisons test.

A significant decrease in RT was observed in 15-month-old DBA/2J mice – it was 35.6% thinner than that of the 3-month-old animals (0.282 ± 0.022 mm vs. 0.182 ± 0.030 mm, respectively P < 0.0001). It was also significantly thinner than in the C57BL/6J mice (0.257 ± 0.042 mm, P < 0.001). However, we noted no significant differences in RT between C57BL/6J and DBA/2J-Gpnmb^+^/SjJ mice at any time. In 18-month-old animals, manganese administration revealed a significantly higher signal enhancement in C57BL/6J mice than in DBA/2J mice (**Figure 4**). No significant changes in the optic nerve diameter were observed.

**Figure 5:**
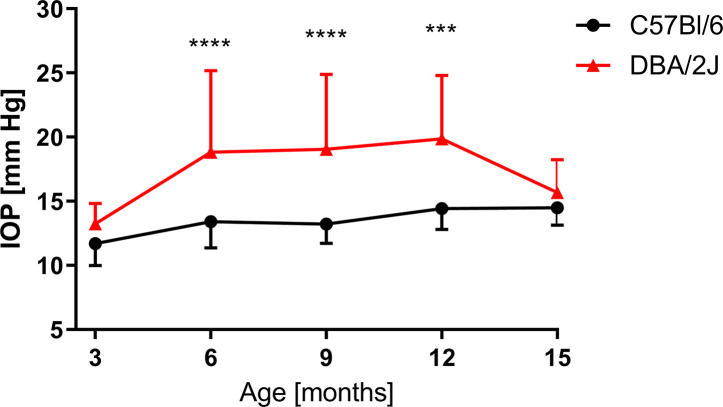
Intraocular pressure (IOP) measured for DBA/2J (3 months, n = 12; 6 months, n = 40; 9 months, n = 34; 12 months, n = 38; 15 months, n = 42), for C57BL/6J (3 months, n = 17; 6 months, n = 27; 9 months, n = 14; 12 months, n = 14; 15 months, n = 19). Two-way ANOVA and *post hoc* Sidak’s test: ***P < 0.001, ****P < 0.0001. Means and SD (for the sake of clarity, error bars are drawn only above, for DBA/2J, or below, for C57Bl/6, the point representing mean values).

Age-dependent changes in the IOP were observed (**Figure 5**). When compared to 3-month-old mice, in 6-month-old DBA/2J mice, the IOP increased by 34.6% (13.3 ± 1.6 mm Hg vs. 18.8 ± 6.4 mm Hg); in 9-month-old mice it increased by 35.9% (19.1 ± 5.8 mm Hg); and it increased 47.5% in 12-month-old mice (19.9 ± 4.9 mm Hg). Between the twelfth and fifteenth months in DBA/2J mice, we observed a decrease in the IOP (15.7 ± 2.6 mm Hg). In C57BL/6J mice, the IOP increased in 12-month-old animals by 25.1% (11.7 ± 1.7 mm Hg in 3-month-old mice vs. 14.4 ± 1.6 mm Hg in 12-month-old mice) and by 23.6% in 15-month-old animals (14.5 ± 1.3 mm Hg) when compared to 3-month-old mice. We analyzed the IOP in 3- and 6-month-old DBA/2J-Gpnmb^+^/SjJ mice and noted no significant differences between those and age-matched C57BL/6J.

**Figure 6:**
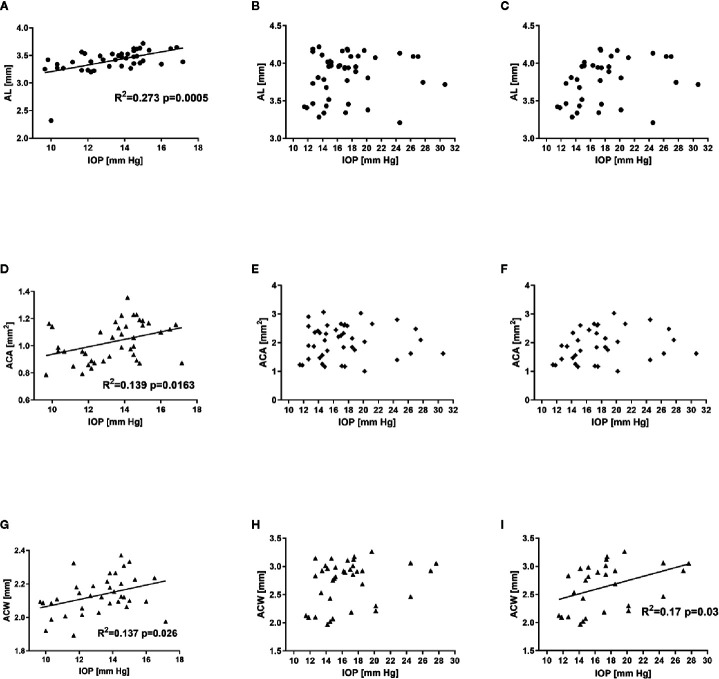
Relationship between intraocular pressure and selected ocular dimensions in C57BL/6J ages 3 to 15 months (first column - **A**, **D**, **G**), DBA/2J mice ages 3 to 15 months (second column – **B**, **E**, **H**) or DBA/2J mice ages 3 to 12 months (third column **C**, **F**, **I**). Graphs illustrate axial length (AL, **A**–**C**), anterior chamber area (ACA, **D**–**F**), and anterior chamber width (ACW, **G**–**I**). Dots represent single values, lines represent linear correlations. Respective R^2^ (if applicable) and P values are placed inside the panels.

The analysis of correlation between various parameters related to ocular morphology and the IOP revealed a significant positive correlation for AL (R^2^ = 0.273, P = 0.0005), ACA (R^2^ = 0.139, P = 0.016), and ACW (R^2^ = 0.137, P = 0.026) in C57BL/6J mice (**Figures 6A, D, G**). On the contrary, such analysis in DBA/2J mice revealed no significant correlations (**Figures 6B, E, H**). However, after excluding the measurements performed in the oldest DBA/2J mice (i.e. analyzing only animals ages 3 to 12 months, **Figures 6C, F, I**), we demonstrated a significant positive correlation between IOP and ACW (R^2^ = 0.17, P = 0.03).

## Discussion

Animal models provide valuable information about the diagnosis and course of disease. They also enable the development of new therapeutic approaches and allow for the monitoring of effects of the therapy (Harada et al., 2019). The DBA/2J mice strain is commonly used as an animal model that mimics many aspects of congenital human glaucoma, particularly pigmentary glaucoma (PG). High IOP in this murine strain is related to the RGC loss and morphological changes of the eyeball and the optic nerve (Schuettauf et al., 2004; Fiedorowicz et al., 2018; Crosbie et al., 2019). Despite the progress in the IOP evaluation methods, performing these measurements in animal models of glaucoma is challenging. In particular, in the DBA/2J mice model, the profound anterior chamber, corneal calcification, and corneal neovascularization or hyphema can significantly impact the accuracy of IOP measurements and optical biometric methods (John et al., 1998; Turner et al., 2017; Harada et al., 2019). For this reason, additional, non-invasive, or low-invasive methods for monitoring the progression of glaucoma in animal models (and in particular in DBA/2J mice) are highly demanded. In this study we used MR techniques that proved to be useful, non-invasive tools for characterization of glaucoma-related changes in the visual pathway,for monitoring the progression of glaucoma, and assessing the efficacy of novel treatment strategies in animal models of glaucoma (Fiedorowicz et al., 2011). Although the spatial resolution of MRI is lower than optical techniques, it has no depth limitations and could be used to conduct a detailed anatomical study of the eyeball and optic nerve, as well as in cases of corneal, lens, or vitreous opacity (Cheng et al., 2006).

Two strains that are not affected by age-related glaucoma-like pathology were used as controls in this study. DBA/2J-Gpnmb^+^/SjJ mice share the genetic background with DBA/2J mice but do not carry one of the mutations that are believed to be responsible for the ocular pathology in DBA/2J mice: *Gpnmb^R150X^*. The presence of functional glycoprotein nonmelanosoma protein B (Gpnmb protein) in DBA/2J-Gpnmb^+^/SjJ mice was shown to prevent the development of iris pigment dispersion (Anderson et al., 2002). However, they carry a mutation in a gene encoding TYRP1 (mutation *Tyrp1^b^*), a major melanosomal glycoprotein and they develop iris stromal atrophy despite no elevation of IOP (Anderson et al., 2002; Howell et al., 2007; Anon, 2020). Another strain used as a control was C57BL/6J that only sporadically develops either easy-to-detect (microphthalmia/anophthalmia) or relatively mild ocular pathologies like corneal opacity. These conditions are believed to be mainly induced by exposure to environmental conditions (Smith et al., 1994). The C57BL/6J strain is also widely used as a control strain for DBA/2J mice (Wong and Brown, 2007; Yang et al., 2018). Not surprisingly, in neither of these strains did we observe a sudden increase in IOP between the third and sixth months of life. The C57BL/6J only displayed a mild, age-dependent IOP elevation that has been previously reported (Cone et al., 2012; Fiedorowicz et al., 2018). Ocular dimensions in both the control strains were also similar.

The progression of glaucoma-related changes in the ocular morphology and IOP in DBA/2J mice, on the other hand, seemed to be more complex. It appears that according to our data we can distinguish three stages of the ocular pathology in DBA/2J mice: (1) around the third month of life; (2) between 6 and 12 months; and (3) >12 months. In 3-month-old animals we noted no differences in the IOP nor in any of the measured ocular dimensions between DBA/2J mice and the control strains; similar results were reported by other groups (Chou et al., 2011). However, it was reported previously that despite no observable IOP elevation, there are some early degenerative processes that may begin around the third month of life in DBA/2J mice, e.g. diminution of anterograde axonal transport within the axons building the optic nerve (Fiedorowicz et al., 2018), reduced spectrin expression (Wilson et al., 2016), RGC apoptosis, degeneration of RGC axons, activation of Müller glial cells (Schuettauf et al., 2004), and microglia activation (Bosco et al., 2015). These neurodegenerative processes seemed, however, to not be advanced enough to be visible on a morphological level in our study.

In DBA/2J mice, between the 6 and 12 months of age, we observed an elevated IOP, which was significantly higher than in C57BL/6J mice. However, in both the control strains, the slope of the age-dependent IOP increase seemed to be similar. On the contrary, while most of the ocular dimensions increased only slightly in the control strains, in DBA/2J mice we observed a steep linear increase in ocular dimensions between the sixth and twelfth months of age. The eye was generally getting bigger as reflected by an increase in axial length and globe area, but it was an effect of alterations mainly of the anterior chamber morphology and, to a smaller extent, the growing of the vitreous chamber (however, the depth of the vitreous chamber remained unchanged). The anterior chamber depth, area, and angle reached significantly higher values in DBA/2J mice than in control strains and these parameters increased in DBA/2J mice between the sixth and twelfth months in a linear manner. Similar observations have been reported in children suffering from primary congenital glaucoma in severe cases, leading to a syndrome called buphthalmos (Hussein et al., 2014; Doozandeh et al., 2017). At this stage of pathology, retinal degeneration was already visible as optic disc cupping visible in T2w images and optic disc cupping was not visible in either of the control strains. Additionally, other groups reported various hallmarks of degeneration at this stage, including decrease of visuomotor function, alterations within the optic nerve, and the optic tract demonstrated by diffusion-weighted MRI (Yang et al., 2018).

Remarkably, we observed a decrease in the IOP between 12 and 15 months of age, similar to the values of those observed in C57BL/6J mice of the same age. Of course, these readings could have been affected by corneal calcification in DBA/2J mice, despite previously reported good agreement regarding the methods for determining IOP measurements involving the TonoLab rebound tonometer that we used in this study and invasive manometric methods (Pease et al., 2011). On the other hand, we noted some changes in the ocular morphology in those mice, which might explain the IOP decrease. Eyeball elongation seemed to stop after the twelfth month of life in DBA/2J mice – neither the axial length nor the globe area differed in animals ages 12 and 15 months. The anterior chamber area and depth also remained stable. However, at this advanced stage of pathology, the vitreous chamber started to be affected. The VCD decreased between the twelfth and fifteenth months of life and in the case of VCA, this effect was noticeable even earlier at the twelfth month of life in DBA/2J. This indicates that after around the twelfth month of life in DBA/2J mice, a disorganization of the eye structure occurs. This might be an effect of iris and ciliary muscular degeneration that fails to sustain the lens; a similar mechanism was postulated in humans (Cheon et al., 2010; Danielewska et al., 2014). In consequence, “pushing” the lens toward the vitreous chamber may, at least partially, compensate for the elevated IOP in the anterior chamber.

Despite the observed IOP decrease in DBA/2J older than 12 months, this stage of pathology seems to be characterized with high dynamics of neurodegenerative processes occurring in the retina and in the optic nerve. The results from our previous study showed a dramatic decrease in anterograde axonal transport within the optic nerve at this stage of pathology (Fiedorowicz et al., 2018) or a decrease in behavioral measures of visual acuity (Wong and Brown, 2007). In this study we demonstrated lower thickness of the retina in DBA/2J mice than in controls – both in T2w anatomical images without enhancement and in T1w images after manganese chloride administration that enhanced the neural retina. However, we did not observe any significant changes in the dimensions of the optic nerve. This could be due to the relatively low size of the optic nerve that results in large measurement errors and in consequent standard deviations.

Finally, we attempted to find ocular dimensions or other parameters that would correlate with the IOP. In mice that developed no glaucoma-like pathology, we found three such parameters: axial length, anterior chamber area, and anterior chamber width. The strongest positive correlation was observed between the AL and the IOP. Despite the results from some studies which have failed to demonstrate a correlation between axial length or other biometric measures of the eye and IOP in patients without glaucoma (Gye et al., 2015), axial length was shown in glaucoma patients to decrease after IOP normalization (Kim et al., 2016). No correlation between any of the biometric parameters in DBA/2J mice was observed – this could be explained by the fact that in the whole population, we included eyes that are affected in different ways – according to the different stages of the pathology that we described above. However, when we excluded 15-month-old mice, there was a significant correlation between ACW and IOP.

Some of the limitations of this study are a consequence of the eye imaging strategy. The use of a surface receive-only coil resulted in the gradual decrease of signal intensity with the distance from the coil. To reduce this effect, the coil size fitted the size of the murine eye and the positioning of the coil was adjusted to provide optimal visualization of all the eyeball structures. However, it is possible that deeper structures like the central retina or the optic nerve are characterized by poorer contrast. Another limitation is possibly imprecise planning of the slices in 2D imaging. In principle, the middle slice of each 2D image was planned to include the optic nerve head and pupil at its widest point. Some mistakes in planning of the middle slice at the image acquisition stage may potentially affect the measurements of the eyeball dimension. However, every image was thoroughly inspected immediately after the acquisition, and in case of any doubts, the measurement was repeated with corrected angles. Relatively low standard deviations in most of the finally calculated morphological parameters suggest that potential mistakes in the planning of slices did not affect these parameters remarkably.

## Conclusion

Our data suggest that morphological changes within the anterior chamber and vitreous chamber could serve as markers of disease progression in animal models of hereditary glaucoma. They correlate with the IOP at the earlier stages of the pathology. However, in contrast to the IOP that could be difficult to measure in older animals, parameters like the anterior chamber area, the anterior chamber depth, or the iridocorneal angle were markedly elevated in animals with advanced pigmentary glaucoma. We propose that these parameters should be included in the evaluation of the efficacy of potential new IOP-lowering drugs in animal models of glaucoma as markers of disease progression and prolonged IOP elevation.

## Data Availability Statement

The raw data supporting the conclusions of this article will be made available by the authors, without undue reservation.

## Ethics Statement

The animal study was reviewed and approved by Warsaw IV Local Ethics Committee.

## Author Contributions

MF designed the research and provided funding, MF, MW-K, JO, and MŚ carried out experiments. MF, MW-K, MŚ, JO, TC, MT, RR, and PB analyzed the experimental results. MF, MW-K, and MT wrote the manuscript, and RR, PB, and PG revised it.

## Funding

This study was supported by Polish National Science Centre grant (DEC-2012/07/D/NZ4/04199) to MF.

## Conflict of Interest

The authors declare that the research was conducted in the absence of any commercial or financial relationships that could be construed as a potential conflict of interest.
